# Association between somatic symptom burden and health-related quality of life in people with chronic low back pain

**DOI:** 10.1371/journal.pone.0193208

**Published:** 2018-02-20

**Authors:** Tomoko Fujii, Hiroyuki Oka, Junji Katsuhira, Juichi Tonosu, Satoshi Kasahara, Sakae Tanaka, Ko Matsudaira

**Affiliations:** 1 Department of Medical Research and Management for Musculoskeletal Pain, 22nd Century Medical & Research Center, Faculty of Medicine, University of Tokyo, Tokyo, Japan; 2 Department of Prosthetics & Orthotics and Assistive Technology, Faculty of Medical Technology, Niigata University of Health and Welfare, Niigata, Japan; 3 Department of Orthopaedic Surgery, Kanto Rosai Hospital, Kanagawa, Japan; 4 Department of Pain and Palliative Medicine, Faculty of Medicine, University of Tokyo, Tokyo, Japan; 5 Department of Orthopaedic Surgery, Faculty of Medicine, University of Tokyo, Tokyo, Japan; Hokkaido Daigaku, JAPAN

## Abstract

Depression is a relevant risk factor for low back pain and is associated with the outcomes of low back pain. Depression also often overlaps with somatisation. As previous studies have suggested that somatisation or a higher somatic symptom burden has a role in the outcomes of low back pain, the aim of the present cross-sectional study was to examine whether somatic symptom burden was associated with health-related quality of life in individuals with chronic low back pain independent of depression. We analyzed internet survey data on physical and mental health in Japanese adults aged 20–64 years with chronic low back pain (n = 3,100). Health-related quality of life was assessed using the EuroQol five dimensions (EQ-5D) questionnaire. Somatic symptom burden and depression were assessed using the Somatic Symptom Scale-8 (SSS-8) and the Patient Health Questionnaire-2 (PHQ-2), respectively. SSS-8 score was categorized as no to minimal (0–3), low (4–7), medium (8–11), high (12–15), and very high (16–32). The association between SSS-8 and EQ-5D was examined using linear regression models, adjusting for depression and other covariates, including age, sex, BMI, smoking, marital status, education, exercise, employment, and the number of comorbid diseases. A higher somatic symptom burden was significantly associated with a lower health-related quality of life independent of depression and the number of comorbid diseases (regression coefficient = 0.040 for SSS-8 high vs. very high and 0.218 for non to minimal vs. very high, p trend <0.0001). In conclusion, somatic symptom burden might be important for the health-related quality of life of individuals with chronic low back pain.

## Introduction

Low back pain (LBP) is a common musculoskeletal health problem. Approximately 80% of people experience LBP at some point during their lifetime [1]. Globally, LBP is the leading cause of years lived with a disability [2]. Therefore, LBP is a significant public health issue.

The etiology of LBP is multifactorial. Individual, physical, and psychosocial factors such as depression are associated with a risk of developing LBP [3–5]. Notably, depression is also a predictor of chronicity [4, 6]. Quality of life is lower in patients with chronic LBP and depression compared to those without depression [7]. Direct health care costs are higher in LBP patients with depression compared to those without depression [8]. Therefore, assessment of depressive symptoms in patients with LBP may be important for predicting prognosis and choosing treatment options in clinical care settings.

Somatisation, which often coexists with depression [9], is defined by Lipowski as “a tendency to experience and communicate somatic distress in response to psychosocial stress and to seek medical help for it” [10]. A systematic review pointed out that most previous studies of pain have used questionnaires on somatic complaints to assess somatisation, which may not account for the full range of the concept of “somatisation” as defined by Lipowski and recommended to use the term “multiple physical symptoms” rather than “somatisation”[11]. Nevertheless, there is some evidence that a higher somatic symptoms burden or “somatisation” is playing some role in LBP. One study reported that the comorbidity of somatic symptoms predicted the development of persistent LBP in the individuals with mild LBP [12]. Another study found that somatisation at baseline, assessed based on the number of medically unexplained symptoms, predicted treatment outcome in patients with LBP [13]. Although depression has been studied in association with LBP, currently, the role of somatic symptom burden in chronic LBP (CLBP) has not been fully explored.

Several self-reported questionnaires have been used to assess common somatic symptoms. A recent systematic review reported that the Patient Health Questionnaire-15 (PHQ-15) and the 12-item Symptom Checklist-90 somatization scale are the most suitable questionnaires for use in large-scale studies [14]. These questionnaires survey relevant symptoms, are relatively short, and have well-established psychometric properties. The Somatic Symptom Scale-8 (SSS-8) was developed as an abbreviated 8-item version of the PHQ-15 [15]. The aim of the present cross-sectional study was to examine whether somatic symptom burden assessed using the SSS-8 is associated with health-related quality of life (HRQoL) in Japanese individuals with CLBP independent of depressive symptoms. We hypothesized that a higher somatic symptom burden is associated with a lower HROoL for patients with CLBP.

## Materials and methods

### Participants

Japanese adults aged between 20–64 years with CLBP (n = 3,100) were included in the present study. The data were acquired from a large internet survey on physical and mental health that was conducted in February of 2015. Participants were recruited by an internet research company, United Inc. (Tokyo, Japan), with which more than 1.37 million individuals across Japan have voluntarily registered. The only inclusion criterion for the survey was an age of 20–64 years. Of the approximately 1.25 million eligible individuals, 270,000 were randomly selected and invited by e-mail to complete the online questionnaire on February 6 2015. We expected that the response rate would be approximately 30% and that approximately 50,000 people would respond within 10 days. The survey was closed on February 16 2015. The questionnaire was configured to automatically reject incomplete responses. All respondents (n = 52,353) gave their consent and were compensated.

A question with an illustration showing the area of pain asked whether a participant had LBP in the past four weeks that may be accompanied with leg pain or numbness, lasted for ≥ one day, and was not related to a menstrual period, pregnancy, or common cold. The following responses were possible: 1) I did not have LBP; 2) I had LBP without difficulty with activities of daily living (ADL); 3) I had LBP with ADL difficulty but without requiring absence from social activities, such as work or school; and 4) I had LBP requiring absence from social activities, such as work or school. Additionally, respondents were asked whether their current LBP lasted for ≥3 months. Individuals with CLBP were defined as those who had LBP with ADL difficulty or sick leave (response 3 or 4 in the first question) which lasted for ≥3 months (affirmative response in the second question), and all respondents with CLBP (n = 3,100) were included in the current analysis. The institutional review board of the University of Tokyo approved this study.

### Assessments

#### Somatic symptom burden

Somatic symptom burden was assessed using the Japanese version of the SSS-8, a self-administered questionnaire. The SSS-8 was developed as an abbreviated 8-item version of the PHQ-15 to assess the presence and severity of common somatic symptoms [15]. The PHQ-15 has been used worldwide [16–21]. The SSS-8 assesses how much the respondent has been bothered by the following somatic symptoms during the past 7 days: 1) stomach or bowel problems; 2) back pain; 3) pain in the arms, legs, or joints; 4) headaches; 5) chest pain or shortness of breath; 6) dizziness; 7) feeling tired or having low energy; and 8) having trouble sleeping. Each item is scored 0 (not at all) to 4 (very much) [22]. The SSS-8 was used as a reference measure in the Diagnostic and Statistical Manual of Mental Disorders (Fifth Edition) (DSM-5) field trials to facilitate the diagnosis of somatic symptom disorder [23]. The German version of the SSS-8 has good reliability and validity for the general German population [22]. We translated the English version of the SSS-8 into Japanese [24]. The Japanese version of the SSS-8 has been linguistically and psychometrically validated [25]. The total SSS-8 score (0–32) was categorized as in Gierk et al. [22] into five groups as follows: no to minimal (0–3); low (4–7); medium (8–11); high (12–15); and very high (16–32).

#### Depressive symptoms

Depressive symptoms were assessed using the Patient Health Questionnaire-2 (PHQ-2), an assessment comprising two questions from the original Patient Health Questionnaire-9 [26]. The questions assess whether the respondent has experienced depression and anhedonia within the past 2 weeks. Although each item is rated on a scale of 0–3 in the original PHQ-2, the present study used the National Center of Neurology and Psychiatry version of the Japanese PHQ-2, which gives each item a binary response of yes or no [27]. Thus, the possible scores for PHQ-2 were 0, 1, or 2.

#### HRQoL

HRQoL was assessed using the 3-level version of the EuroQol five dimensions (EQ-5D-3L) questionnaire that measures general health status [28]. The EQ-5D contains five questions assessing mobility, self-care, usual activities, pain/discomfort, and anxiety/depression [28]. Responses are converted into a single index score of general health status ranging from −0.11 to 1.00; a score of 1 indicates perfect health and a score of 0 indicates death. The Japanese version of the EQ-5D-3L has been approved by the EuroQol Group and is widely used in research [29]. Because the present study was based on the internet survey, we did not assess the visual analog scale (EQ-VAS).

#### Covariates

Information on age, gender, body weight, height, marital status, education, employment status, and current smoking status were collected through a self-administered questionnaire. The respondents were asked to choose one response for marital status, education and employment status: current marital status (1. married, 2. never married, 3. divorced and 4. widowed), education background (1. middle school, 2. high school, 3. vocational school, 4. higher professional school, 5. college, 6. undergraduate, 7, graduate, and 8. other), and employment status (1. regular employee, 2. part-time worker, 3. temporary worker, 4. business executives, 5. family business worker, 6. home worker, 7. student, 8. housewife or husband, 9. without an occupation, and 10 other). Smoking status was asked using a single question “Have you ever smoked ≥100 cigarettes or been smoking for ≥6 months and smoked sometimes or every day during the past month?” Participants were asked whether they performed exercises regularly over the past year, such as walking and jogging that lasted for ≥30 minutes. The possible responses were none, 1–2 times per month, once per week, or more than twice per week. Having regular exercise was defined as exercising more than twice per week. Participants were asked to answer whether they were seeking treatment for the following chronic conditions with yes/no response: hypertension, heart disease, dyslipidemia, lung disease, diabetes, gastrointestinal disease, renal disease, liver disease, anemia/hematological disease, thyroid disease, cancer, gynecological disease, urological disease, skin disease, sleep apnea, other otolaryngological disease, eye disease, dental problems, osteoarthritis, headache, rheumatoid arthritis, fibromyalgia, osteoporosis, obesity, and others. Two questions very similar to those about LBP asked whether the respondent had knee pain. Body mass index (BMI) was calculated based on the self-reported body weight and height as weight (kg)/height (m)^2^.

### Statistical analysis

Initially, the characteristics of the participants were examined using descriptive statistics such as the mean and percentage and were compared between PHQ-2 groups using Kruskal-Wallis test for continuous variables and chi-square test for categorical variables. To assess the crude correlation between SSS-8 total scores and EQ-5D scores, Spearman correlation coefficient was estimated. To examine the association between somatic symptom burden assessed using SSS-8 and EQ-5D score, linear regression models were used. Because we assumed that relatively high somatic symptom burden would be more problematic rather than one score change in SSS-8, and because of possible non-linear association, we used five categories for SSS-8 scores as the primary independent variable. Model 1 was a crude model which included only SSS-8 as the independent variable. Model 2 included SSS-8 and depression (as measured using the PHQ-2) simultaneously. An interaction between the SSS-8 and PHQ-2 was not statistically significant. Model 3 was further adjusted for age (continuous), sex, and BMI. Model 4 was further adjusted for lifestyle and individual factors: smoking status (yes/no), marital status (married or other), education (≥college degree or other), regular exercise (yes/no), and employment status (regular employee or other). Model 5, the final model, was further adjusted for the number of comorbid diseases (0–25). The p-value for linear trend for the association between SSS-8 and EQ5D was obtained by treating five SSS-8 categories as the ordinal variable. These potential confounders, which would be associated with both somatic symptom burden and EQ5D, were chosen a priori. We did not use methods such as stepwise selection for model building, because these methods could lead to overfitted models and data-driven results. Multicollinearity was not suspected, with all variance inflation factors (VIFs) being < 2. A stratified analysis by sex and age was conducted exploratory. The participants were relatively homogenous in terms of age (20–64). Therefore, we split the participants into two groups: those younger than 50 and those older than 50 years. Significance of interaction by sex, age, or sex-age group was tested. Analyses were conducted using SAS version 9.4 (SAS Institute, Inc., Cary, NC, USA). All analyses were two-sided and an α-level of 0.05 was considered statistically significant.

## Results

The characteristics of the participants are shown in Table 1. The mean age of the participants was 44.5 ± 11.2 and 48% were women. The PHQ-2 score was 0 in 1,576 (51%) participants, 1 in 632 (20%), and 2 in 892 (29%). In these individuals with chronic LBP, 20.6% answered they had chronic knee pain. The mean EQ-5D score was 0.78 ± 0.18, and this score decreased as the PHQ-2 score increased. Mean SSS-8 score was 9.67 ± 6.68. As the PHQ-2 score increased, the proportion of individuals with a very high SSS-8 score increased.

**Table 1:** Characteristics of the participants with chronic low back pain.

	All	PHQ-2 = 0	PHQ-2 = 1	PHQ-2 = 2	*p-value
	(n = 3100)	(n = 1576)	(n = 632)	(n = 892)	
Age, mean (SD)	44.5 (11.2)	45.8 (11.0)	44.5 (11.5)	42.1 (11.1)	< .0001
Women, n (%)	1483 (47.8)	743 (47.1)	311 (49.2)	429 (48.1)	0.669
BMI, n (%)					0.018
<25	2333 (75.3)	1184 (75.1)	484 (76.6)	665 (74.6)	
25–29	589 (19.0)	320 (20.3)	103 (16.3)	166 (18.6)	
≥30	178 (5.7)	72 (4.6)	45 (7.1)	61 (6.8)	
Current smoking, n (%)					0.0004
Yes	1064 (34.3)	489 (31.0)	233 (36.9)	342 (38.3)	
No	2036 (65.7)	1087 (69.0)	399 (63.1)	550 (61.7)	
Marital status, n (%)					< .0001
Not married	1327 (42.8)	544 (34.5)	299 (47.3)	484 (54.3)	
Married	1773 (57.2)	1032 (65.5)	333 (52.7)	408 (45.7)	
Education, n (%)					0.043
No college	1650 (53.2)	804 (51.0)	350 (55.4)	496 (55.6)	
Some college	1450 (46.8)	772 (49.0)	282 (44.6)	396 (44.4)	
Regular exercise, n (%)					0.001
No regular exercise	2487 (80.2)	1222 (77.5)	524 (82.9)	741 (83.1)	
Regular exercise	613 (19.8)	354 (22.5)	108 (17.1)	151 (16.9)	
Employment status, n (%)					0.0001
Non regular employee	1829 (59.0)	876 (55.6)	411 (65)	542 (60.8)	
Regular employee	1271 (41.0)	700 (44.4)	221 (35)	350 (39.2)	
Number of comorbid disease, mean (SD)	1.2 (2.0)	1.0 (1.8)	1.3 (2.0)	1.4 (2.3)	< .0001
Chronic knee pain	639 (20.6)	239 (15.2)	148 (23.4)	252 (28.3)	< .0001
EQ-5D, mean (SD)	0.78 (0.18)	0.84 (0.16)	0.75 (0.17)	0.70 (0.17)	< .0001
SSS-8 items, n (%)^†^					
Stomach or bowel problems	944 (30.5)	349 (22.1)	197 (31.2)	398 (44.6)	< .0001
Back pain	2192 (70.7)	1052 (66.8)	476 (75.3)	664 (74.4)	< .0001
Pain in arms, legs, or joints	1336 (43.1)	573 (36.4)	301 (47.6)	462 (51.8)	< .0001
Headaches	1004 (32.4)	383 (24.3)	206 (32.6)	415 (46.5)	< .0001
Chest pain or shortness of breath	570 (18.4)	161 (10.2)	133 (21)	276 (30.9)	< .0001
Dizziness	616 (19.9)	205 (13)	130 (20.6)	281 (31.5)	< .0001
Feeling tired or having low energy	1464 (47.2)	475 (30.1)	340 (53.8)	649 (72.8)	< .0001
Trouble sleeping	1077 (34.7)	326 (20.7)	251 (39.7)	500 (56.1)	< .0001
SSS-8, n (%)					< .0001
No to minimal (0–3)	590 (19.0)	445 (28.2)	77 (12.2)	68 (7.6)	
Low (4–7)	785 (25.3)	488 (31.0)	152 (24.1)	145 (16.3)	
Medium (8–11)	616 (19.9)	297 (18.9)	156 (24.7)	163 (18.3)	
High (12–15)	505 (16.3)	186 (11.8)	122 (19.3)	197 (22.1)	
Very high (16–32)	604 (19.5)	160 (10.2)	125 (19.8)	319 (35.8)	

*p-value based on a chi-square test or Kruskal-Wallis test.

†Those who were bothered at least somewhat.

Abbreviations: SD, standard deviation; BMI, body mass index; EQ-5D, EuroQol Five Dimension; SSS-8, the Somatic Symptom Scale-8

SSS-8 total score and EQ5D score were significantly negatively correlated (Spearman correlation coefficient = -0.55, p-value<0.0001). The results of the regression models are shown in Table 2. In the crude model, SSS-8 categories were significantly associated with EQ-5D. EQ-5D scores were lower in those who had higher SSS-8 scores. With the adjustment for PHQ-2, regression coefficients for SSS-8 were attenuated greater than 10%, but the SSS-8 categories were significantly associated with EQ-5D scores independent of the PHQ-2 score. The PHQ-2 score was also independently associated with EQ-5D. Adjustment for demographic, lifestyle and other individual variables did not change the estimates essentially. In the final multiple model adjusted for the number of the comorbid conditions, the regression coefficients for SSS-8 were further attenuated but the association was still significant. Individuals with CLBP and a higher SSS-8 score had lower EQ-5D scores (regression coefficient β = 0.040 for SSS-8 high vs. very high, and β = 0.218 for non to minimal vs. very high, p-trend<0.0001).

**Table 2:** Association between EQ-5D and SSS-8 from linear regression models.

	Model 1			Model 2			Model 3			Model 4			Model 5		
Parameters	β	SE	p-value	β	SE	p-value	β	SE	p-value	β	SE	p-value	β	SE	p-value
**Intercept**	0.649	0.006	< .0001	0.695	0.007	< .0001	0.777	0.013	< .0001	0.812	0.014	< .0001	0.815	0.014	< .0001
**SSS-8**^**†**^															
No to minimal	0.274	0.009	< .0001	0.242	0.009	< .0001	0.239	0.009	< .0001	0.234	0.009	< .0001	0.218	0.009	< .0001
Low	0.184	0.008	< .0001	0.160	0.008	< .0001	0.159	0.008	< .0001	0.155	0.008	< .0001	0.142	0.008	< .0001
Medium	0.125	0.009	< .0001	0.109	0.009	< .0001	0.111	0.009	< .0001	0.109	0.009	< .0001	0.098	0.009	< .0001
High	0.056	0.009	< .0001	0.049	0.009	< .0001	0.048	0.009	< .0001	0.047	0.009	< .0001	0.040	0.009	< .0001
Very high	reference			reference			reference			reference			reference		
**PHQ-2**															
0				reference			reference			reference			reference		
1				-0.048	0.007	< .0001	-0.049	0.007	< .0001	-0.043	0.007	< .0001	-0.042	0.007	< .0001
2				-0.068	0.007	< .0001	-0.073	0.007	< .0001	-0.067	0.007	< .0001	-0.066	0.007	< .0001

SSS-8^†^: no to minimal (0–3); low (4–7); medium (8–11); high (12–15); very high (16–32)

Model 1: Crude model

Model 2: PHQ-2 adjusted

Model 3: Further adjusting for age, sex, and body mass index

Model 4: Further adjusting for smoking, marital status, education, regular exercise, and employment status

Model 5: Further adjusting for the number of chronic diseases

β, coefficient; SE, standard errors; SSS-8, the Somatic Symptom Scale-8; PHQ-2, Patient Health Questionnaire-2

The results of the stratified analysis by sex, age and sex-age group are shown in Table 3. The results were consistent with those in the overall sample. Although the p-values for high SSS-8 in men aged ≥50 year and women aged ≥50 years were >0.05, this could be due to the small sample size in these groups. No significant interaction by age, sex or age-sex categories was found.

**Table 3:** Association between EQ-5D and SSS-8 from linear regression models by sex and age.

		Men			Women			Total		
	Parameters	β	SE	p-value	β	SE	p-value	β	SE	p-value
**Age <50 years**	**Intercept**	0.799	0.031	< .0001	0.860	0.027	< .0001	0.837	0.021	< .0001
	**SSS-8**^**†**^									
	No to minimal	0.229	0.018	< .0001	0.200	0.016	< .0001	0.214	0.012	< .0001
	Low	0.156	0.016	< .0001	0.124	0.014	< .0001	0.140	0.011	< .0001
	Medium	0.107	0.017	< .0001	0.084	0.014	< .0001	0.097	0.011	< .0001
	High	0.052	0.017	0.003	0.036	0.013	0.008	0.044	0.011	< .0001
	Very high	reference			reference			reference		
			n = 996			n = 938			n = 1934	
**Age ≥50 years**	**Intercept**	0.917	0.087	< .0001	0.836	0.076	< .0001	0.887	0.058	< .0001
	**SSS-8**^**†**^									
	No to minimal	0.229	0.023	< .0001	0.207	0.021	< .0001	0.224	0.015	< .0001
	Low	0.147	0.022	< .0001	0.135	0.018	< .0001	0.145	0.014	< .0001
	Medium	0.106	0.022	< .0001	0.084	0.019	< .0001	0.098	0.014	< .0001
	High	0.038	0.025	0.123	0.018	0.019	0.353	0.031	0.015	0.044
	Very high	reference			reference			reference		
			n = 621			n = 545			n = 1166	
**Total**	**Intercept**	0.782	0.021	< .0001	0.833	0.019	< .0001			
	**SSS-8**^**†**^									
	No to minimal	0.229	0.014	< .0001	0.205	0.012	< .0001			
	Low	0.153	0.013	< .0001	0.131	0.011	< .0001			
	Medium	0.108	0.013	< .0001	0.088	0.011	< .0001			
	High	0.047	0.014	0.001	0.032	0.011	0.003			
	Very high	reference			reference					
			n = 1617			n = 1483				

SSS-8^†^: no to minimal (0–3); low (4–7); medium (8–11); high (12–15); very high (16–32)

Adjusted for PHQ-2, continuous age, sex (when not be stratified), body mass index, smoking, marital status, education, regular exercise, employment status, and the number of chronic diseases

β, coefficient; SE, standard errors; SSS-8, the Somatic Symptom Scale-8; PHQ-2, Patient Health Questionnaire-2

## Discussion

We found that a higher somatic symptom burden was associated with lower EQ-5D scores independent of depressive symptoms and the number of comorbid diseases in Japanese individuals with CLBP. The results were consistent across sex and age. The difference in EQ-5D between SSS-8 high vs. very high was 0.04 and was 0.22 for SSS-8 non to minimal vs. very high among all participants. Yoshizawa et al. reported that the minimal clinically important change (MCIC) of EQ-5D in Japanese with chronic noncancer pain was 0.10 [30]. Soer et al. reported a MCIC of EQ-5D as 0.03 in patients with chronic LBP in Netherland [31]. According to these studies, the differences in EQ-5D between SSS-8 groups would be clinically meaningful. Our result suggests that somatic symptom burden is important for the quality of life of individuals with CLBP.

Somatisation is defined by Lipowski as “a tendency to experience and communicate somatic distress in response to psychosocial stress and to seek medical help for it” [10]. Somatisation, depression, and anxiety are frequent mental disorders in primary health care, and often overlap in patients [9]. Depression has been studied extensively, and it is established that depression is a predictor of the chronicity of LBP [4, 6]. Somatisation has also been studied for LBP and other pain research. Nonetheless, the assessment of “somatisation” is problematic. Crombez et al. noted in their systematic review that most previous studies of pain have used questionnaires on somatic complaints to assess somatisation, which may not account for the full range of the concept of “somatisation” as defined by Lipowski, such as whether symptoms were not explained by pathological findings and whether the individual was attributing the symptom to physical illness and seeking medical help for it. The authors recommended using the term“multiple physical symptoms” rather than “somatisation”[11]. The authors also warn that somatisation scores could be elevated artificially by the somatic symptom that is the primary complaint, such as LBP.

Nevertheless, there is some evidence that somatic symptoms burden or “somatisation” is associated with HRQoL. Somatisation, depression, and anxiety can coexist, but each of them may have an independent role in HRQoL. Lowe et al. reported that depression, anxiety, and somatisation were each independently associated with Short-Form General Health Survey (SF-20) scores in primary clinic patients, although the effect size of each was only small to moderate [9]. In this study, somatisation was defined as a PHQ-15 score of ≥ 15. The authors discussed that using this high threshold would reflect somatisation and not just somatic symptom severity. A review of nine population-based studies found that total somatic symptom scores were associated with healthcare use and predicted health status independent of depression, anxiety, and a number of general medical illnesses [32]. We adjusted for the counts of chronic conditions as in this review study to try to consider the comorbid disease and to reflect how much individuals were bothered by somatic symptoms, which is one nature of somatisation. Our study results were consistent with these previous studies, showing that somatic symptom burden is associated with HRQoL independent of depression and the number of comorbid diseases in a group of individuals with CLBP.

Previous studies suggest that somatisation has a role in the outcomes of LBP [6]. Matsudaira et al. reported that the comorbidity of somatic symptoms, as assessed using the brief job stress questionnaire (BJSQ), was associated with the development of persistent LBP in urban Japanese workers with mild LBP [12]. In patients with LBP that were treated by chiropractors, somatisation at baseline that was assessed using the Four Dimensional Symptom Questionnaire was associated with pain intensity, functional status, and perceived recovery [33]. In hospital patients with LBP, baseline somatisation based on the number of symptoms for which physicians could find no clear cause assessed using the Screening for Somatoform Disorders Questionnaire, was correlated with HRQoL (MOS 36-Item Short-Form Health Survey (SF-36) scores) at follow-up, and was inversely associated with ≥ 50% reduction in pain one-year after surgical or conservative treatment [13]. These studies suggest the possibility that somatic symptom burden would lead to a worse LBP outcome and result in a lower HRQoL. However, these studies do not always adjust for depression. Depression is a risk factor for the onset and chronicity of LBP [4, 6], and somatisation often coexists with depression. Therefore, it might be difficult to determine whether somatic symptom burden predicts LBP outcomes independent of depression.

There is another possible explanation for our study results. Individuals with LBP often suffer from multisite pain or other musculoskeletal disorders [34–36], which could result in higher somatic symptom assessment scores. Previous studies show that as the number of pain sites increase, functional ability or HRQoL decreased [35, 36]. Although we did not assess pain in another body site other than LBP or knee pain, 43% of the participants answered that they were bothered at least somewhat by “pain in arms, legs, or joints” in the SSS-8. In addition, studies have showed that chronic conditions such as fibromyalgia, chronic LBP, irritable bowel syndrome, temporomandibular joint disorder, interstitial cystitis, chronic fatigue syndrome, and headache are linked by central sensitization and called functional somatic syndromes (FSS) [37–39]. The FSS conditions may overlap in one patient [39]; therefore, it is possible that individuals with more disabling chronic LBP suffered from other musculoskeletal disorders and/or FSS symptoms that decreased HRQoL in these individuals.

A strength of the present study is its large sample size with information on relevant covariables. The participants were not recruited in clinical settings; therefore, the possibility of selection bias due to seeking treatment should be low. The independent variable and the outcome were assessed using the validated tools, which would reduce the risk of classification bias. However, there are a few limitations of the present investigation. First, depression was assessed using only two questions; thus, misclassification is possible, which would be non-differential. Second, measures of anxiety were not available. Anxiety also often overlaps with somatisation and is associated with HRQoL [9]; thus, residual depression and anxiety may have confounded the results. This could have contributed to the overestimation, but the magnitude is unknown. Third, we did not collect the information on the pain in other sites than LBP and knee pain. Finally, the participants of the present study were recruited online and thus our results are not necessarily representative of the Japanese population.

In conclusion, somatic symptom burden might be an important factor for HRQoL in individuals with CLBP independent of depressive symptoms and the number of chronic conditions.

## Supporting information

S1 File Data set. Data set for this analysis.
(CSV)

S2 File STROBE check list. STROBE checklist for cross-sectional studies.
(DOC)
